# One-Month Rifapentine–Isoniazid Regimen Versus Six-Month Isoniazid Monotherapy for Latent Tuberculosis: Experience from a Reference Center

**DOI:** 10.3390/medicina62030542

**Published:** 2026-03-14

**Authors:** Joana Marques Simões, Dalila Ferreira, Teresa Mourato, Ana Pais, André Dias, Margarida Torres, Luís Coelho

**Affiliations:** 1 CDP Dr. Ribeiro Sanches, Pulmonary Department, Santa Maria Health Local Unit, 1649-045 Lisbon, Portugal; 2 Comprehensive Health Research Centre (CHRC), NOVA Medical School, NOVA University of Lisbon, 1099-085 Lisbon, Portugal

**Keywords:** latent tuberculosis infection, isoniazid, rifapentine plus isoniazid, adverse drug reactions, hepatotoxicity, treatment completion rate

## Abstract

*Background and Objectives*: Isoniazid monotherapy has been the most widely used treatment for latent tuberculosis infection (LTBI). Although effective, it has been associated with poor adherence and a higher incidence of adverse events. The shorter duration of rifamycin-based regimens has become increasingly preferable. The one month of daily rifapentine plus isoniazid (1HP) has demonstrated low toxicity and higher completion rates in HIV-infected populations. This study aims to compare the completion rate and adverse events between the 1HP and daily isoniazid for 6 months (6H) regimens in the non-HIV adult population. *Materials and Methods*: Retrospective, observational, longitudinal study, followed at the National Reference Center for Tuberculosis (Lisbon, Portugal), from January 2024 to January 2025. Treatment-related symptoms and liver function were assessed throughout the treatment. Relevant hepatic toxicity was defined as aspartate transaminase (AST) and/or alanine transaminase (ALT) > 1.5 times the upper limit of normal (ULN). *Results*: A total of 90 and 74 patients were assigned to the 1HP and 6H groups, respectively. No significant differences were observed in the frequency of reported adverse symptoms between the 1HP and 6H groups (28.9% vs. 23.0%, *p* = 0.4). The 1HP regimen was associated with a significantly lower risk of relevant hepatic toxicity (4.6% vs. 32.9%, *p* < 0.001) and a higher rate of treatment completion (97.8% vs. 67.6%, *p* < 0.001). Adverse drug reactions were the leading cause of treatment discontinuation in both groups, with hepatic toxicity and gastrointestinal intolerance being the most frequent events. A therapeutic switch to rifampicin was required in 16.2% of patients receiving the 6H regimen, whereas no switch was needed in the 1HP group. *Conclusions*: The 1HP regimen was associated with a higher rate of treatment completion and lower hepatic toxicity with no significant differences in the reported adverse symptoms.

## 1. Introduction

Despite being preventable and usually curable, tuberculosis (TB) remains the leading cause of mortality from a single infectious agent worldwide, accounting for approximately 10.8 million incident cases and 1.25 million deaths in 2023. Of these, approximately 410,000 were patients with multidrug-resistant tuberculosis (MDR-TB), and although constituting only 5% of the total burden of TB, the mortality of these cases is substantially higher, contributing to 15–20% of global TB mortality [1]. About one-fourth of the global population is estimated to have latent tuberculosis infection (LTBI), and the TB preventive treatment (TPT) has emerged as a major public health priority to reduce the risk for developing active disease [2,3]. Despite advances, global coverage of TPT remains insufficient to achieve the targets of the World Health Organization (WHO) End TB Strategy [1]. Furthermore, maximizing regimen completion and minimizing adverse events are also critical. The isoniazid monotherapy has historically been the standard regimen for TB prevention, with well-documented efficacy in reducing the risk of progression to active TB disease by 60% to 90% [2,4]. However, the shorter rifamycin-based regimens have become increasingly preferred due to their equivalent efficacy, improved adherence rates, and lower incidence of hepatotoxicity [5,6,7,8,9,10]. The WHO currently recommends all these regimens for TB prevention regardless of HIV status, including six or nine months of daily isoniazid (6H or 9H), three months of daily isoniazid plus rifampicin (3HR), three months of weekly isoniazid plus rifapentine (3HP), and, as alternative options, four months of daily rifampicin (4R) and 1HP [2]. The new and shorter 1HP regimen has previously demonstrated effectiveness and higher treatment completion for TPT in HIV-infected populations when compared to the standard 9H regimen [10]. However, no comparative studies have been conducted between the 1HP and isoniazid monotherapy regimens in non-HIV-infected individuals, particularly with the 6H regimen.

This study aims to compare adverse drug reactions and treatment completion rates between the 1HP and standard 6H regimen in non-HIV-infected individuals with LTBI.

## 2. Materials and Methods

### 2.1. Study Design and Population

From January 2024 to January 2025, we conducted this retrospective, observational, longitudinal study at the National Tuberculosis Reference Center in Lisbon, Portugal. Eligible patients were aged ≥18 years and had a positive tuberculin skin test (TST) and/or Interferon-γ release assay (IGRA; measured using QuantiFERON-TB Gold) for LTBI in the context of documented exposure to a confirmed case of active TB identified through contact investigation. All patients treated either with 1HP or 6H in the study period were included. Patients with suspected or confirmed active TB, as determined by clinical symptoms or chest findings, were excluded. Additional exclusion criteria included individuals with documented exposure to cases of drug-resistant TB, known allergy to isoniazid or rifapentine, pregnant or breastfeeding women, immunosuppressive conditions, and candidates for transplantation or biological therapy. Data were collected from clinical and laboratory records.

### 2.2. Protocol

The 1HP group received directly observed therapy with daily isoniazid 300 mg plus rifapentine 600 mg for a total of 28 doses. By contrast, the 6H group was treated with self-administered daily isoniazid at a dose of 5 mg/kg/day (up to a maximum of 300 mg) for 182 doses. Treatment was considered complete when all scheduled doses were taken.

### 2.3. Safety Monitoring

All patients in the 6H group had medical follow-up and laboratory tests at the beginning, after 15 days, and then monthly. In the 1HP group, the follow-up was performed at the beginning and after 15 days. At every visit, treatment-related symptoms and adherence were assessed. Patients were instructed to self-report side effects between follow-up visits. Additionally, relevant hepatic toxicity was considered as an elevation of AST and/or ALT greater than 1.5× the ULN. Patients who presented AST and/or ALT 3–5× ULN and >5× ULN were discontinued from TPT. Patients who presented AST and/or ALT 1.5 times the ULN were maintained under strict surveillance and discontinued from TPT if they became symptomatic or presented a rising level of AST and/or ALT.

### 2.4. Statistical Methods

Continuous variables were compared using the *t*-test or Wilcoxon rank-sum test, as applicable. Categorical variables were assessed with Pearson’s chi-squared test or Fisher’s exact test when expected counts were <5. Associations between predictors and the outcome were examined using a logistic regression model adjusted for baseline covariates (age, gender, and presence of comorbidities), and results are reported as odds ratios (OR) with 95% confidence intervals (CI). Unadjusted estimates were not presented unless the statistical significance of the results changed between adjusted and unadjusted estimates. All tests were two-sided, with *p* < 0.05 considered statistically significant. All statistical analyses were conducted using R statistical software (version 4.3.0; R Core Team, 2023).

## 3. Results

### 3.1. Study Population

A total of 164 eligible participants were enrolled in the study, with 90 assigned to the 1HP group and 74 to the 6H group. The mean age was 39 years in the 1HP group and 49 years in the 6H group. Male participants predominated in the 1HP group, whereas females were more common in the 6H group. Comorbidities were generally infrequent (11.1%), with cardiovascular diseases and diabetes being the most prevalent (Table 1). IGRA was performed in all patients, and no TST was conducted.

### 3.2. Adverse Events

Transaminase data were available for 157 of the 164 participants. Relevant hepatic toxicity occurred significantly less frequently in the 1HP group than in the 6H group (4.6% vs. 32.9%, *p* < 0.001; Table 2). The odds of developing hepatic toxicity were 10.4 times higher in participants receiving the 6H regimen (OR 10.4, 95% CI, 3.41–40.1). In most cases, ALT and/or AST levels were elevated 1.5–3 times above the ULN, whereas elevations exceeding five times the ULN were observed in 8.6% of those treated with 6H. No cases of acute liver failure or death were recorded.

The overall frequency of adverse symptoms did not differ significantly between groups (28.9% vs. 23.0%, *p* = 0.4). More than one symptom was reported by 8.9% of participants in the 1HP group and by 2.7% in the 6H group. Gastrointestinal symptoms were the most frequently reported adverse event in both groups (Table 1). Though a wide CI often reflects precision limitations related to a low event count, which is common in studies of adverse events and may reduce statistical precision for that specific outcome, the OR remains the best estimate of effect in our studies. It should be noted that an adjustment for multiple comparisons was not applied in our primary analysis, since our study design focused on a single, pre-specified primary endpoint (adverse events).

### 3.3. Treatment Completion Rate and Switch to Alternative Regimens

The overall treatment completion rate was 84.1%, significantly higher in the 1HP group than in the 6H group (97.8% vs. 67.6%, *p* < 0.001; Table 3), possibly associated with direct supervision and shorter regimen duration. Participants receiving the 6H regimen had 96% lower odds of completing treatment (OR 0.04, 95% CI, 0.01–0.17).

Adverse drug reactions were the main reason for treatment discontinuation in both groups. In the 1HP group, both discontinuations were attributed to gastrointestinal intolerance, whereas in the 6H group, most discontinuations were due to hepatotoxicity (54.2%), followed by voluntary withdrawal (33.3%). A therapeutic switch to the 4R regimen was performed in 16.2% of participants in the 6H group, whereas no treatment modifications occurred in the 1HP group. Permanent treatment discontinuation without transition to another regimen occurred in participants who opted to withdraw by personal decision. Since assessing potential interactions between key variables in the regression model is crucial for analyzing clinically relevant relationships such as age and comorbidities, we performed an effect modification analysis. However, there was no strong evidence of a meaningful interaction effect observed.

## 4. Discussion

Diagnosing and treating LTBI is a fundamental step in reducing future cases of TB and is one of WHO’s main strategies for TB control and mortality reduction. On average, 5–10% of infected patients will develop tuberculosis disease, most within the first 5 years after initial infection, with immunocompromised individuals at greatest risk [11,12]. Therefore, identifying and treating at-risk groups is crucial to reducing the emergence of new cases of the disease. People living with HIV, individuals in contact with TB patients, people in settings such as prisons and homeless shelters, and those with immunodeficiency conditions (e.g., cancer, under immunosuppressive therapies, chronic renal failure, and diabetes) are at high risk of TB disease and are hence priority groups for receiving TPT.

Between 2018 and 2022, 15.5 million people were provided with TPT, distributed as 11.3 million people with HIV, 2.2 million children under 5 years of age, and 2 million people aged ≥5 years. In September 2023, at the second United Nations high-level meeting (UNHLM) on TB, Member States endorsed a political declaration committing them to, by the end of 2027, having provided 45 million individuals with TPT to protect them from developing TB disease during this period [13]. Therefore, it is essential to have treatments available that, in addition to being short, are inexpensive and have few adverse effects.

Adverse drug reactions and treatment duration remain key concerns in the use of TPT, with potential implications for adherence, treatment completion, and the effectiveness of the current standard isoniazid regimen. In this context, the shorter duration of rifamycin-based regimens has shown a clear advantage, contributing to their growing preference over isoniazid monotherapy [7,8,9].

Rifapentine, a semisynthetic derivative of rifampicin developed in 1965, exhibits favorable pharmacological and microbiological properties, including a long half-life and greater potent bactericidal activity against *Mycobacterium tuberculosis*. These characteristics have established rifapentine as a key component in the treatment of both active TB and LTBI [2,14,15]. The 3HP regimen has demonstrated efficacy and safety comparable to the 9H regimen, while achieving substantially higher treatment completion rates (82.1% vs. 69.0%) [7,16]. These findings were corroborated in different population settings, including HIV-infected and uninfected adults, adolescents, and children as young as two years old. Conversely, the effectiveness of 1HP has mainly been established in the HIV-infected population. It has been demonstrated to be noninferior to the standard 9H regimen for preventing TB and death in adults and adolescents older than 13 years old living with HIV, with a higher completion rate (97% vs. 90%) and a comparable safety profile [10]. Although the studies available at the time were conducted in HIV-infected populations, given the promising results and the pharmacological potential of rifapentine, the WHO has endorsed 1HP as a preventive strategy for both HIV-infected and uninfected individuals [2]. More recently, a study conducted in non-HIV-infected participants reported comparable adherence (82.9 vs. 84.5%) and safety outcomes between the 1HP and 3HP regimens [17].

Since our experience with 1HP was limited, we wanted to evaluate the frequency of liver function test abnormalities in this regimen compared to 6H. To do this, we stratified the severity into 3 groups (1.5–3× ULN, 3–5× ULN, and >5× ULN), considering the two groups with the highest values as patients with hepatotoxicity relevant to treatment discontinuation, as used in most studies. In these two groups (3–5× ULN and >5× ULN), the 6H regimen showed a higher number of patients with liver toxicity. Our study demonstrated that patients receiving the 1HP regimen also achieved a higher treatment completion rate and a lower incidence of hepatotoxicity compared with those treated with the 6H regimen. The higher completion rate observed with 1HP (97.8% vs. 68.0%) is likely attributable to directly observed therapy and the shorter duration of treatment. Moreover, the low completion rate observed among patients receiving the 6H regimen may reflect real-world outcomes rather than those reported in randomized trials. Consistent with previous reports [7,10,17,18,19], hepatotoxicity was more frequent among patients receiving 6H and accounted for most treatment discontinuations. The proportion of participants who experienced adverse symptoms attributed to the study drug did not differ significantly between the two groups. Gastrointestinal intolerance was the most common adverse event in the 1HP group, contrasting with the systemic drug reactions described in earlier studies, particularly urticaria and flu-like syndrome [7,10,17].

This study has several limitations. First, data were obtained from medical records and may have been subject to incomplete or missing information. Second, the sample size was relatively small, especially when we consider the several sub-groups. Nevertheless, this study complements previous research by providing real-world evidence on the effectiveness of the 1HP regimen for TPT in a broader population, using the shorter isoniazid regimen as a comparator. However, it should be highlighted that the 1 HP treatment was performed under direct observation, contributing to greater adherence and increasing the difference in adherence observed to the self-administered 6H regimen. Third, children, adolescents, and pregnant or breastfeeding women were excluded. Consequently, the findings may not be generalizable to these populations. Also, a cost-effectiveness analysis was not done, and the follow-up period did not permit the evaluation of the possibility of late hepatotoxicity of the 1HP regimen. Finally, like other rifamycins, rifapentine acts as an inducer of cytochrome P-450 enzymes and the P-glycoprotein efflux transporter, thereby increasing the potential for pharmacokinetic drug–drug interactions [7,20,21]. Therefore, it would be important to investigate such interactions, although the low frequency of comorbidities in both groups likely resulted in minimal concomitant medication use.

## 5. Conclusions

In conclusion, to the best of our knowledge, this is the first comparative study between the 1HP and 6H regimens for the treatment of LTBI in non-HIV infected individuals. The 1HP regimen demonstrated higher completion rates and lower hepatotoxicity in this real-world cohort. However, causal inference should be interpreted with caution, given the observational design and baseline imbalances. The imbalance between the groups results from the fact that patients selected for the 1HP regimen came from homeless institutions and other at-risk groups, as well as schools, where it was easier to administer treatment through direct observation, while patients in the 6H regimen were from the general population identified for TPT in index case screenings and were therefore older; in these cases the usual practice is self-administration. We acknowledge that this strategy contributed to better treatment adherence in the 1HP regimen group. However, our approach is an effective strategy for ensuring adherence to TPT, especially when we need to treat large groups of patients living in closed communities.

Further studies are warranted to corroborate and extend these results in larger and more diverse populations, including children less than 13 years old, pregnant and breastfeeding women, and individuals with different clinical settings, such as those eligible for transplantation or biologic therapy. Furthermore, more robust cost-effectiveness studies are needed to guide population-specific preventive therapy guidelines. Finally, ensuring universal availability and affordability of rifapentine-based regimens will be essential to maximize their impact on global TB prevention efforts.

## Figures and Tables

**Table 1:** Characteristics of the participants.

	1HP (n = 90)	6H (n = 74)	Total (n = 164)	*p*-Value ^1^
**Age—yr**				<0.001
Mean (SD)	39 (16)	49 (16)	44 (17)	
Median (Min; Max)	35 (19; 72)	48 (19; 84)	41 (19; 84)	
**Sex—no. (%)**				<0.001
Male	65 (72.2)	31 (41.9)	96 (58.5)	
Female	25 (27.8)	43 (58.1)	68 (41.5)	
**Patients reporting comorbidities—no. (%)**	4 (4.4)	14 (18.9)	18 (11.0)	0.006
Arterial Hypertension	0 (0)	3 (4.1)	3 (1.8)	
Atrial fibrillation	0 (0)	1 (1.4)	1 (0.6)	
Ischemic heart disease	0 (0)	2 (2.7)	2 (1.2)	
Diabetes	0 (0)	4 (5.4)	4 (2.4)	
Asthma	1 (1.1)	2 (2.7)	3 (1.8)	
Malignancy	1 (1.1)	1 (1.4)	2 (1.2)	
Chronic hepatitis	1 (1.1)	1 (1.4)	2 (1.2)	
Gilbert Syndrome	1 (1.1)	0 (0)	1 (0.6)	

^1^ *t* test; Pearson’s chi-squared test; Fisher’s exact test.

**Table 2:** Reported adverse drug reactions during treatment period.

	1HP (n = 87)	6H (n = 70)	Total (n = 157)	*p*-Value ^1^
**Relevant Hepatic Toxicity—no. (%)**	4 (4.6)	23 (32.9)	27 (17.2)	<0.001
1.5–3× ULN	3 (3.4)	14 (20.0)	17 (10.8)	
3–5× ULN	1 (1.1)	3 (4.3)	4 (2.5)	
>5× ULN	0 (0)	6 (8.6)	6 (3.8)	
	**1HP** **(n = 90)**	**6H** **(n = 74)**	**Total** **(n = 164)**	***p*** **-value ^1^**
**Patients reporting symptoms—no (%)**	26 (28.9)	17 (23.0)	43 (26.2)	0.4
Nausea	13 (14.4)	3 (4.1)	16 (9.6)	
Vomiting	2 (2.2)	3 (4.1)	5 (3.0)	
Anorexia	0 (0)	1 (1.4)	1 (0.6)	
Abdominal pain	3 (3.3)	1 (1.4)	4 (2.4)	
Gastroesophageal reflux	3 (3.3)	0 (0)	3 (1.8)	
Malaise	1 (1.1)	0 (0)	1 (0.6)	
Fatigue	3 (3.3)	1 (1.4)	4 (2.4)	
Myalgia	2 (2.2)	1 (1.4)	3 (1.8)	
Arthralgia	0 (0)	1 (1.4)	1 (0.6)	
Cutaneous Rash	0 (0)	1 (1.4)	1 (0.6)	
Cutaneous pruritus	0 (0)	2 (2.7)	2 (1.2)	
Weight loss	0 (0)	1 (1.4)	1 (0.6)	
Headache	6 (6.7)	2 (2.7)	8 (4.9)	
Insomnia	1 (1.1)	0 (0)	1 (0.6)	
Numbness	0 (0)	1 (1.4)	1 (0.6)	
Visual disturbances	0 (0)	1 (1.4)	1 (0.6)	
Menstrual cycle disturbances	1 (1.1)	0 (0)	1 (0.6)	
Oligoanuria	1 (1.1)	0 (0)	1 (0.6)	

^1^ Fisher’s exact test.

**Table 3:** Treatment completion rate and causes of treatment discontinuation.

	1HP (n = 90)	6H (n = 74)	Total (n = 164)	*p*-Value ^1^
**Treatment completion rate—no. (%)**	88 (97.8)	50 (67.6)	138 (84.1)	<0.001
**Switch to Rifampicin—no. (%)**	0 (0)	12 (16.2)	12 (7.3)	<0.001
**Cause of discontinuation—no. (%)**				0.03
Hepatotoxicity	0 (0)	13 (54.2)	13 (50.0)	
Gastrointestinal intolerance	2 (100)	2 (33.3)	4 (15.4)	
Cutaneous rash	0 (0)	1 (1.5)	1 (3.8)	
Participant’s decision	0 (0)	8 (33.3)	8 (30.8)	

^1^ Fisher’s exact test.

## Data Availability

The data presented in this study are available on request from the corresponding author. The data are not publicly available due to privacy restrictions.

## Abbreviations

The following abbreviations are used in this manuscript:

1HP	One-month daily rifapentine plus isoniazid
3HP	Three months of weekly isoniazid plus rifapentine
3HR	Three-month daily isoniazid plus rifampicin
4R	Four-month daily rifampicin
6H	Six-month daily isoniazid
9H	Nine-month daily isoniazid
ALT	Alanine aminotransferase
AST	Aspartate aminotransferase
CI	Confidence intervals
HIV	Human immunodeficiency virus
IGRA	Interferon-γ release assay
LTBI	Latent tuberculosis infection
OD	Odds ratio
TB	Tuberculosis
TPT	Tuberculosis preventive treatment
TST	Tuberculin skin test
UNHLM	United Nations high-level meeting
ULN	Upper Limit of Normal
WHO	World Health Organization
